# Letter to the editor: Long-term survival after female pelvic organ-sparing radical cystectomy versus standard radical cystectomy: a multi-institutional propensity score-matched analysis

**DOI:** 10.1097/JS9.0000000000000869

**Published:** 2023-11-23

**Authors:** Yingjie Chen, Yanling Liao, Yuwei Yang, Xiaochun Zheng

**Affiliations:** a Department of Anesthesiology, Shengli Clinical Medical College of Fujian Medical University, Fujian Provincial Hospital; b Department of Anesthesiology and Perioperative Medicine, Fuzhou General Hospital of Nanjing Military Command Area: 900th Hospital of PLA; c Fujian Emergency Medical Center, Fujian Provincial Key Laboratory of Emergency Medicine, Fujian Provincial Key Laboratory of Critical Care Medicine, Fujian Provincial Co-Constructed Laboratory of “Belt and Road”, Fuzhou, People’s Republic of China

*Dear Editor*,

With great interest, we read the recent study ‘Long-term survival after female pelvic organ-sparing radical cystectomy versus standard radical cystectomy: a multi-institutional propensity score-matched analysis’ by Zhong *et al*.^1^. The authors conducted a large, multicenter, retrospective study to compare the long-term survival of standard radical cystectomy (RC) with pelvic organ-sparing radical cystectomy (POPRC). No significant difference in long-term survival was found among female patients undergoing the two surgical methods. These noteworthy findings could be of great value in the treatment and management of female bladder cancer patients.

The association between POPRC and patients’ long-term survival has been subject to inconclusive findings. To address this critical challenge, the authors used a propensity-matched analytic model that included sex, age, previous illness, tumor characteristics, and perioperative variables, which effectively minimized the influence of potential confounders. However, several issues remain, and we would appreciate the authors’ insights and comments.

RC is the gold standard treatment for patients with muscle-invasive bladder cancer (MIBC) and high-risk non-muscle-invasive bladder cancer. However, adjuvant treatments (including neoadjuvant therapy and adjuvant radiotherapy) are equally important for long-term survival^2^. Therefore, we consider the potential impact on the results of the model lacking a portion of the data.

With respect to adjuvant radiotherapy, patients with POPRC usually have related limited preoperative cancer invasion, and due to the potential impairment of preserved organs by radiation therapy, physicians may consider other alternatives when developing an adjuvant regimen. Thus, there may be a difference in the proportion of patients undergoing POPRC who undergo adjuvant radiotherapy compared with patients undergoing RC. Furthermore, although the role of adjuvant radiotherapy on long-term survival is currently controversial^3^, there are more supportive reports. The results of a recent phase II trial showed that the addition of postoperative adjuvant radiotherapy to chemotherapy in patients with locally advanced disease and negative margins significantly improved local relapse-free survival^4^. The effects of adjuvant radiotherapy on long-term prognosis should not be completely ignored, and this omission in the model may introduce bias into the results.

With regard to neoadjuvant therapy, the authors mentioned the limitations of the study in the Discussion section. We concur that neoadjuvant chemotherapy has a benefit in downstaging and improving long-term survival^5^. The application of neoadjuvant therapy may have been an important confounding factor, and the omission of neoadjuvant therapy may potentially lead to misdirection.

In conclusion, this study is important evidence supporting the application of POPRC in female patients. Future utilization of a more complete database and consideration of the recommendations will provide rigor and quality of the study and improve our understanding of POPRC.

## Ethical approval

This article does not require any human or animal subjects to acquire such approval.

## Consent

This article does not require any human or animal subjects to acquire such approval.

## Sources of funding

The authors received no financial support.

## Author contribution

X.Z.: conceptualized, reviewed, and edited; Y.C., Y.L., and Y.Y.: writing and made the draft and updated it. All authors have critically reviewed and approved the final draft and are responsible for the content and similarity index of the manuscript.

## Conflicts of interest disclosure

The authors declare no conflicts of interest.

## Research registration unique identifying number (UIN)

None.

## Guarantor

All authors.

## Data availability statement

This study did not have any data analysis. The datasets utilized and/or analyzed in this study can be obtained from the corresponding author upon a reasonable request.

## Provenance and peer review

Not commissioned, externally peer-reviewed.
